# Association of Changes of lifestyle behaviors before and during the COVID-19 pandemic with mental health: a longitudinal study in children and adolescents

**DOI:** 10.1186/s12966-022-01327-8

**Published:** 2022-07-26

**Authors:** Mi Xiang, Yujie Liu, Shohei Yamamoto, Tetsuya Mizoue, Keisuke Kuwahara

**Affiliations:** 1 Ministry of Education—Shanghai Key Laboratory of Children’s Environmental Health, School of Public Health, Shanghai Jiao Tong University School of Medicine, Chongqing road n.227, Shanghai, 200025 China; 2 School of Public Health, Shanghai Jiao Tong University School of Medicine, Chongqing road n.227, Shanghai, 200025 China; 3 Department of Epidemiology and Prevention, National Center for Global Health and Medicine, Tokyo, 162-8655 Japan; 4 Teikyo University Graduate School of Public Health, 2-11-1 Kaga, Itabashi-ku, Tokyo, 173-8605 Japan

**Keywords:** COVID-19, Children and adolescents, Physical activity, Screen time, Mental health, Change of lifestyle behavior

## Abstract

**Background:**

We examined the prospective associations of changes in lifestyle behaviors before/during the COVID-19 pandemic, namely physical activity and screen time, with mental health. Furthermore, the impacts of physical activity and screen time on mental health during the pandemic were examined cross-sectionally.

**Methods:**

A two-wave longitudinal study was conducted among 2423 children and adolescents in Shanghai, China. Lifestyle behavior variables (physical activity and screen time) and psychological variables (depressive symptoms, anxiety, and stress) were measured using a self-reported questionnaire in January and March 2020. A series of multivariable logistic regressions were performed to examine the associations between changes in lifestyle behaviors in two waves and psychological problems. The combined associations of physical activity and screen time with psychological problems were also explored using the second wave data.

**Results:**

Compared to students with persistently short screen time before and during the COVID-19 pandemic, those with prolonged screen time (OR = 1·36 for depression, OR = 1·48 for anxiety) and those with persistently long screen time (OR = 1·70 for depression, OR = 2·13 for anxiety) reported a higher risk of psychological symptoms. The association between changes in physical activity and psychological symptoms was not statistically significant after adjustment for demographic factors, socioeconomic status, and screen time. During the COVID-19 pandemic, engaging in longer screen time (OR = 1·44 for depression, OR = 1·55 for anxiety) was associated with worsened psychological conditions, while engaging in increased physical activity (OR = 0·58 for depression, OR = 0·66 for anxiety) was associated with better psychological conditions.

**Conclusions:**

Our study suggests that promoting physical activity and limiting leisure screen time during the COVID-19 pandemic are important to prevent and mitigate psychological problems in children and adolescents. Therefore, effective interventions targeting lifestyle behaviors are needed to protect children and adolescents’ physical and mental health.

**Supplementary Information:**

The online version contains supplementary material available at 10.1186/s12966-022-01327-8.

## Introduction

The ongoing spread of coronavirus disease 2019 (COVID-19) has posed tremendous challenges worldwide. Governments have enforced several quarantine and restriction orders as emergency measures. School closures implemented to prevent the disease from spreading have led to undesirable outcomes. Despite the well-intentioned starts, more than 150 million children and adolescents in 165 countries have been affected by the closures [1], raising concerns and objections against the acts [2, 3].

Lifestyle behaviors, including physical activity and screen use, are crucial determinants of both physical and mental health in children and adolescents [4–7]. Studies have shown that participating in physical activity and reducing screen time may increase social interaction and social support [8, 9]. However, evolving evidence has revealed significantly decreased physical activity levels and prolonged screen time during home quarantine, [10] potentially impacting students’ mental health. Moreover, accumulating data suggest that the lifestyle habits and negative psychological status acquired during the pandemic may extend into adulthood [11–15]. Therefore, a more comprehensive insight into the relationship between changing lifestyle behaviors and mental health during the pandemic is urgently needed to assist policy and intervention development and implementation by governments, schools, health professionals, and parents to protect children and adolescents worldwide.

Our previous study has revealed the substantial impacts of the COVID-19 pandemic on children and adolescents’ lifestyle behaviors [16]. However, to the best of our knowledge, no quantitative, prospective studies have examined the association of dramatic changes in physical activity or screen time with mental health among children and adolescents during the current COVID-19 pandemic. Therefore, the present study aimed to 1) investigate the associations of changes in physical activity and screen time with mental health among children and adolescents during COVID-19; 2) examine the combined association of physical activity and screen time with mental health during the pandemic.

## Methods

### Study design and participants

A web-based short-term longitudinal study was conducted among a sample of children and adolescents (Grades 1–9) and their parents in Shanghai, China. Fourteen districts of Shanghai were the primary sampling units (16 districts of Shanghai in total including two suburbs – Qingpu and Chongming) and were all invited to the survey. Seven of them agreed to participate in the survey. In seven designated districts, we randomly selected 1 to 2 schools from each district by cluster sampling. Four primary schools, five junior high schools, and one school integrating primary and junior high schools participated in the first survey, and five of them completed the first to the second wave. All the children and adolescents in the selected schools and their parents were invited.

The first survey was conducted among 7544 children and adolescents in 10 schools as well as their parents (approximately 83% participation rate) between January 3rd and 21st, 2020. A public health emergency was announced in Shanghai on January 24th, 2020. A questionnaire, containing questions on lifestyle habits and mental health status of children and adolescents, was implemented. The second survey was administered between March 13th and 23rd, 2020 (approximately two months after the COVID-19 outbreak, right before the public health emergency was downgraded in Shanghai on March 24th, 2020) to 3042 children, adolescents, and their parents (approximately 93% participation rate) in five schools who participated in the first survey. The two online surveys for students and parents were distributed by head teachers to each parent. The survey also obtained demographic characteristics and socioeconomic status of the parents.

Research assistants and teachers were invited to answer any related questions from students and parents concerning the survey to ensure the accuracy of the responses. Moreover, parents were instructed to assist primary school students. Students and parents were permitted to answer the questions only after consenting to study participation. The study was approved by the Ethics Committee of Shanghai, Jiao tong University School of Medicine (SJUPN- 201813). Informed consent was obtained from all parents.

Of the included 2423 children and adolescents, 1241 were boys (51·2%]) and 1182 were girls (48.8%), ranging in age from 6 to 17 (mean [SD] age, 11·7 [2·3] years) were extracted. Participants who completed all two surveys and provided valid data on variables were included. Four students were excluded due to invalid answers of 24 hours physical activity time or screen time. A flow chart of participant exclusion is shown in Supplementary Fig. 1.

### Measurement of physical activity and screen time

Physical activity was defined as body movement supported by skeletal muscle contraction that required energy expenditure above basal levels. Moderate-and vigorous-intensity physical activities were measured using the Global Physical Activity Questionnaire (GPAQ), developed by the World Health Organization [17] and validated in children and adolescents by previous studies [18, 19]. According to the Physical Activity Guidelines Advisory Committee, we defined inactive as no engagement in moderate-and vigorous-intensity physical activity, and active as engagement in moderate-and vigorous-intensity physical activity [20]. Changes in physical activity were determined by the physical activity level (active or inactive) of the first and second surveys, yielding four categories.

Leisure screen time was reported by participants using the number of days per week and the time spent per day on watching TV/videos (DVD, video app, etc.); internet use (news, douban, etc.); computer/smartphone games; and social platform use (QQ, WeChat, etc.) during leisure times. We further categorized leisure screen time into two groups, short (≤2 h/day) and long (> 2 h/day), according to the guidelines for children [21]. Changes in leisure screen time were defined by the combinations of screen time levels (short or long) in the first and second surveys.

### Measurement of mental health

Mental health status during the COVID-19 pandemic was assessed using a Chinese version of the 21-item Depressive Anxiety Stress Scales (DASS21) [22, 23]. DASS-21 contains 21 items: 7 items for depressive symptoms, 7 items for anxiety, and 7 items for stress. Each item has four response options corresponding to different extent of depression, anxiety, and stress state, ranging from 0 (did not apply to me at all) to 3 (applied to me very much or most of the time). Participants were asked to score their state of the past week. Thus, the DASS-21 yields separate depressive symptoms, anxiety symptoms, and stress subscale scores (0–21). This questionnaire has previously been validated in children and adolescents [23] and was sensitive to cultural and linguistic issues. Its reliablity and validity has been assessed for Chinese students [24]. Scores for depressive symptoms, anxiety, and stress were calculated by summing the scores of the relevant items and multiplying them by two [25, 26]. The participants were determined as having depressive symptoms, anxiety, and stress if the subscale score exceeded 9, 7, and 14, respectively.

Additionally, students were asked to report their depressive symptoms and anxiety levels using the Children’s Depressive Symptoms Inventory-Short Form (CDI-S) [27] and Social Anxiety Scale for Children (SASC) in the first survey (baseline) [28]. The CDI-S consisted of 10 items, each having 3 response options, on the extent to which participants had experienced each state over the past week, yielding a total score of between 0 and 20, with a higher score indicating more depressive symptomatology. The SASC also consisted of 10 items, where participants were asked how often each of the 10 items was true for them (“never so,” “sometimes so,” and “always so”), yielding a total score of between 0 and 20. A higher score indicated that a participant perceived a higher level of social anxiety. Depressive symptoms and anxiety in the first survey were defined by scores of more than 7 and 8, respectively.

### Other measurements

Sociodemographic variables included gender, grade, maternal and paternal education, and family income. To ensure accuracy, family information, such as maternal and paternal education and family income were reported by parents. We used data from the first survey for the present analyses.

### Statistical analyses

Characteristics of children and adolescents according to their physical activity level or screen time level in the first survey were expressed in percentages. In the main analysis, we examined the impact of the changes in physical activity level and screen time before and during the COVID-19 pandemic on mental health (depressive symptoms, anxiety, and stress) in the second survey using logistic regression. In model 1, we adjusted for students’ age, gender, grade, and psychological problems collected in the first survey (data of 11 students with missing or invalid data of ages or grade were excluded). In model 2, we adjusted for paternal education, maternal education, and family income in addition to the factors adjusted in model 1 (data of 121 parents were further excluded due to missing or invalid data on education and family income). In model 3, we further mutually adjusted for physical activity (active or inactive) and leisure screen time (shorter or longer) collected in the first survey. The goodness-of-fit of the models was evaluated through the Hosmer-Lemeshow test.

We also created models to examine the joint impact of physical activity and screen time on mental health in the second survey. In model 1, we adjusted for students’ age, sex, grade, paternal education, maternal education, and family income. In model 2, we adjusted for physical activity or leisure screen time collected in the second survey in addition to the factors adjusted in model 1. In model 3, we adjusted for factors in model 1 and physical activity, screen time, and psychological problems obtained in the first survey. Since data on perceived stress was not available in the first survey, the multivariable models of stress were different from those for depression and anxiety. Data were analyzed using IBM SPSS Statistics, version 26. *P*-values (two-sided) less than 0.05 were considered statistically significant.

## Results

### Descriptive statistics

Before the COVID-19 pandemic, only 153 (6·3%) students did not participate in any moderate-to vigorous-intensity physical activity, and 176 (7·3%) had long leisure screen time (> 2 h/day). Table 1 showed the characteristics of 2423 students according to physical activity or screen time level in the first survey. The characteristics of physically active students were similar to the inactive group. Compared with students with short leisure screen time, students with long screen time tended to be more anxious and older. The characteristics of the 2423 students according to physical activity or screen time level acquired in the second survey are shown in Supplementary Table 1. There were no significant difference in sociodemographic variables between the retained five schools and the excluded five schools.

**Table 1:** Sample Characteristics according to physical activity or screen time level at the first survey (*n* = 2423)

	Overall	Physical activity		*P* value	Leisure screen time		*P* value
		Active^a^	Inactive		Short^b^	Long	
Students (*n*)	2423	2270	153		2247	176	
Gender							
Girls	48.8	48.4	54.2	0.162	48.5	52.3	0.336
Boys	51.2	51·6	45·8		51·5	47·7	
Grade							
1–3	23.6	23·4	26·8	0.475	24·8	8·0	<.001
4–6	35.4	35·7	31·4		35·8	30·1	
7–9	41.0	40·9	41·8		39·3	61·9	
Educational attainment							
Father							
Middle school or below	7.4	7·6	4·8	0.360	7·0	12·7	0.009
High school	82.8	82·6	86·4		82·8	82·5	
University/College	7.4	7·6	5·4		7·8	3·0	
Master or higher	2.4	2·3	3·4		2·4	1·8	
Mother							
Middle school or below	9.8	10·0	6·8	0.624	9·4	15·7	0.028
High school	84.3	84·1	87·8		84·6	81·3	
University/College	4.2	4·2	4·1		4·4	1·8	
Master or higher	1.7	1·7	1·4		1·7	1·2	
Family income (CNY)							
< 100,000	12.0	11·9	13·6	0.758	11·5	18·7	0.016
100,000 to 200,000	29.4	29·4	29·3		29·4	30·1	
> 200,000 to 400,000	33.3	33·2	34·7		33·4	31·3	
> 400,000	16.3	16·3	16·3		16·9	9·6	
No answer	9.0	9·2	6·1		8·9	10·2	
Psychological problems							
Depression	15.6	15·7	15·0	0.830	14·8	26·1	<.001
Anxiety	47.6	47·0	57·5	0.011	46·6	60·8	<.001

Data are shown as % unless otherwise specified

There were missing data for Grade (*n* = 1), Educational attainment (*n* = 121), and Family income (*n* = 121), they were excluded from the calculation of each proportion

^a^ Inactive was defined as no moderate- to vigorous-intensity physical activity (0 min per week), whereas active was defined as some moderate- to vigorous-intensity physical activity (> 0 min per week)

^b^ Short time was defined as ≤2 hours per day whereas long time was defined as > 2 hours per day

### Effect of physical activity or leisure screen time on psychological problems during the COVID-19 pandemic

During the pandemic, the prevalence of physically inactive (no moderate- and vigorous-intensity activity) students increased from 6·3% to 28·2%, while those with sufficient activity (≥60 min/day) substantially decreased from 60·0% to 17·7%. Meanwhile, leisure screen time was prolonged, and 30·9% of students engaged in longer screen times for leisure in the second survey (Supplementary Fig. 2). Psychological problems were prevalent in the second survey: 18·0, 23, and 14% of children and adolescents had depressive symptoms, anxiety, and stress, respectively (Supplementary Table 1).

**Fig. 1:**
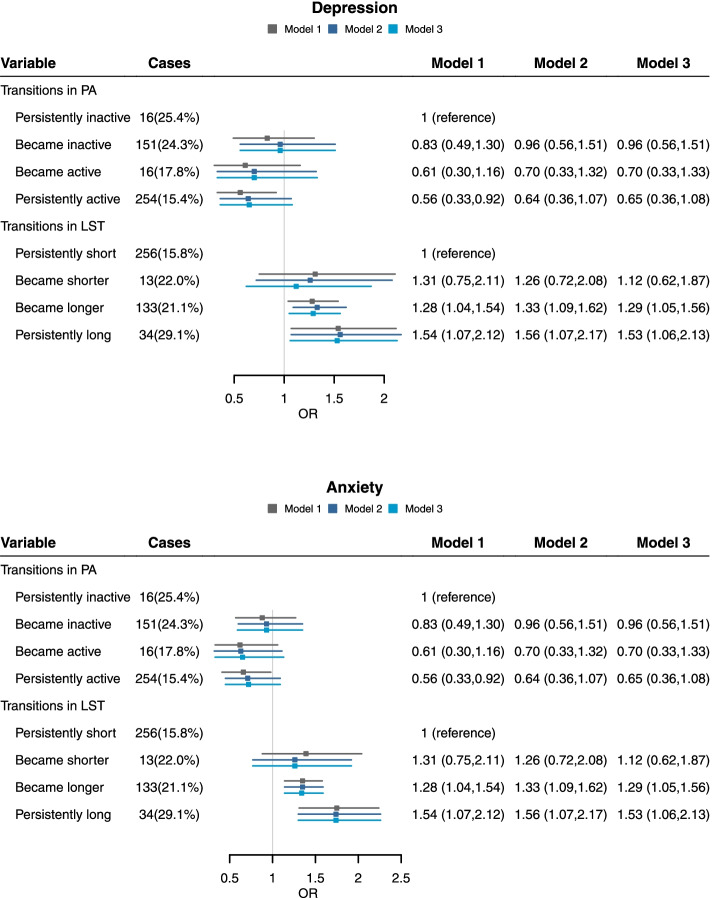
Associations of changes in physical activity or leisure screen time before and during COVID-19 pandemic with psychological conditions (*n* = 2423). PA: Physical activity; LST: Leisure screen time. Data are shown as odds ratios (95% confidence intervals) of each psychological problem in the second survey calculated using logistic regression. Model 1Adjusted for student’s age, gender, grade, and psychological problem in the first survey of each outcome (e.g., for analysis of depression, depression level in the first survey was adjusted for, but anxiety and stress were not). Model 2 Further adjusted for paternal education, maternal education, and family income. Model 3 Mutually adjusted for physical activity (active or inactive) and leisure screen time (shorter or longer). Children with missing or invalid data of age were excluded in model 1 (*n* = 11). Children with missing or invalid data of parental education or income were further excluded in models 2 and 3 (*n* = 121)

The relationship between changes in physical activity or screen time before and during the COVID-19 pandemic with mental health in the second survey was shown in Table 2 and Fig. 1. Longitudinal changes in leisure screen time before and during the COVID-19 pandemic were significantly associated with depressive symptoms and anxiety. As compared with persistently short screen time, prolonged screen time and persistently long screen time were associated with severer depressive symptoms after adjustment for physical activity (model 3); the corresponding adjusted odds ratios (ORs) (95% confidence intervals [CIs]) were 1·36 (1·06, 1·75) and 1·70 (1·07, 2·71), respectively. A similar tendency was shown for anxiety. The Hosmer-Lemeshow test showed that the models were well-fitted (*p* > 0.05). The association of changes in physical activity with psychological symptoms was not statistically significant after adjustment for demographic factors, socioeconomic status, and screen time (models 2 and 3). Similar results were observed for anxiety.

**Table 2:** Associations of changes in physical activity or leisure screen time before and during COVID-19 pandemic with psychological conditions (*n* = 2423)

	Cases	OR^a^	*P* value^d^	OR^b^	*P* value^d^	OR^c^	*P* value^d^
Depression							
Change in physical activity							
Persistently inactive (*n* = 63)	16 (25·4%)	1 (reference)					
Became inactive (*n* = 620)	151 (24·3%)	0·78 (0·42,1·44)	0.019	0·95 (0·49,1·83)	0.594	0·95 (0·49,1·83)	0.807
Became active (*n* = 90)	16 (17·8%)	0·54 (0·24,1·22)		0·63 (0·27,1·48)		0·64 (0·27,1·50)	
Persistently active (*n* = 1650)	254 (15·4%)	**0·49 (0·27,0·89)**		0·57 (0·30,1·10)		0·58 (0·30,1·11)	
Change in leisure screen time							
Persistently short (*n* = 1616)	256 (15·8%)	1 (reference)					
Became shorter (*n* = 59)	13 (22·0%)	1·39 (0·72,2·67)	0.065	1·33 (0·68,2·62)	0.267	1·14 (0·58,2·24)	0.525
Became longer (*n* = 631)	133 (21·1%)	**1·34 (1·05,1·71)**		**1·42 (1·11,1·83)**		**1·36 (1·06,1·75)**	
Persistently long (*n* = 117)	34 (29·1%)	**1·71 (1·09,2·68)**		**1·74 (1·09,2·79)**		**1·70 (1·07,2·71)**	
Anxiety							
Change in physical activity							
Persistently inactive (*n* = 63)	21 (33·3%)	1 (reference)					
Became inactive (*n* = 620)	182 (29·4%)	0·83 (0·47,1·47)	0.271	0·90 (0·50,1·64)	0.414	0·90 (0·49,1·63)	0.098
Became active (*n* = 90)	19 (21·1%)	0·52 (0·25,1·10)		0·53 (0·24,1·17)		0·55 (0·25,1·20)	
Persistently active (*n* = 1650)	337 (20·4%)	**0·56 (0·32,0·97)**		0·62 (0·35,1·12)		0·63 (0·35,1·14)	
Change in leisure screen time							
Persistently short (*n* = 1616)	317 (19·6%)	1 (reference)					
Became shorter (*n* = 59)	18 (30·5%)	1·53 (0·85,2·73)	0.420	1·35 (0·73,2·47)	0.314	1·35 (0·73,2·47)	0.398
Became longer (*n* = 631)	176 (27·9%)	**1·47 (1·18,1·84)**		**1·48 (1·18,1·85)**		**1·48 (1·18,1·85)**	
Persistently long (*n* = 117)	47 (40·2%)	**2·14 (1·42, 3·21)**		**2·13 (1·40,3·26)**		**2·13 (1·40,3·26)**	

Data are shown as odds ratios (95% confidence intervals) of each psychological problem in the second survey calculated using logistic regression

^a^ Adjusted for student’s age, gender, grade, and psychological problem in the first survey of each outcome (e.g., for analysis of depression, depression level in the first survey was adjusted for, but anxiety and stress were not)

^b^ Further adjusted for paternal education, maternal education, and family income

^c^ Mutually adjusted for physical activity (active or inactive) and leisure screen time (shorter or longer)

Children with missing or invalid data of age were excluded in model 1 (*n* = 11). Children with missing or invalid data of parental education or income were further excluded in models 2 and 3 (*n* = 121)

^d^ P Value was conducted for the Hosmer-Lemeshow test

**Fig. 2:**
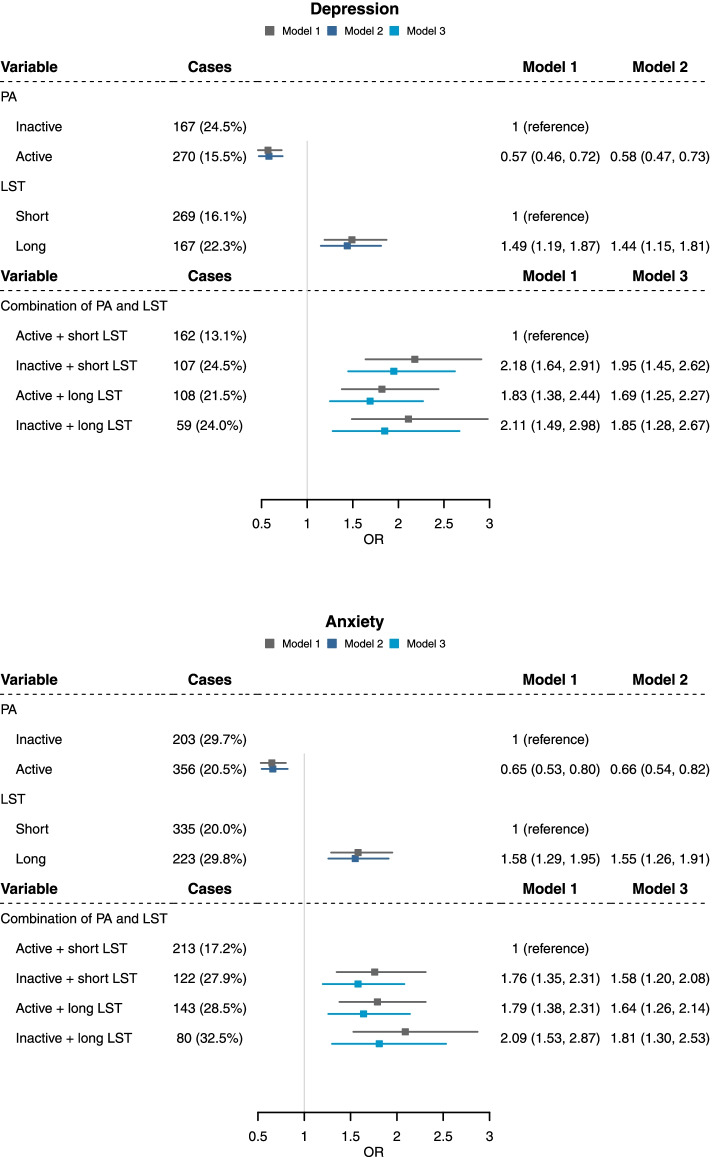
Associations of physical activity and leisure screen time with psychological conditions during COVID-19 pandemic (*n* = 2426). PA: Physical activity; LST: Leisure screen time. Data are shown as odds ratios (95% confidence intervals) of having psychological problems in the second survey calculated by logistic regression. Model 1 Adjusted for baseline age, sex, grade, paternal education, maternal education, and family income. Model 2 Additionally, and mutually adjusted for physical activity or leisure screen time in the second survey. Model 3 Adjusted for factors in model 1 and physical activity, screen time, and psychological problems in the first survey. Children with missing or invalid data of age were excluded in model 1 (*n* = 11). Children with missing or invalid data of parental education or income were further excluded in models 2 and 3 (*n* = 121)

Figure 2 shows the impact of physical activity and leisure screen time on mental health during the COVID-19 pandemic. Physically active children and adolescents during the COVID-19 pandemic had a lower odds of depressive symptoms (OR: 0·58, 95% CI: 0·47–0·73) and anxiety (OR: 0·66, 95% CI: 0·54–0·82), whereas students with longer screen time had a higher odds of depressive symptoms (OR: 1·44, 95% CI: 1·19–1·87) and anxiety (OR: 1·55, 95% CI: 1·26–1·91) (in model 2). Joint association of physical activity and screen time showed that, as compared with active students with shorter leisure screen time, other groups had a higher odds of depressive symptoms (OR: 1·85, 95% CI: 1·28–2·67 for inactive + long leisure screen time; OR: 1·69, 95% CI: 1·25–2·27 for active + long screen time; OR: 1·95, 95% CI: 1·45–2·62 for inactive + short leisure screen time) and anxiety (OR: 1·81, 95% CI: 1·30–2·53 for inactive + long leisure screen time; OR: 1·64, 95% CI: 1·26–2·14 for active + long screen time; OR: 1·58, 95% CI: 1·20–2·08 for inactive + short leisure screen time) in model 3.

## Discussion

To the best of our knowledge, this is the first large-population based longitudinal study to examine the prospective associations of changes in physical activity or screen time before and during the COVID-19 pandemic with mental health. Additionally, this is the first study to examine the single and joint associations of physical activity or screen time in relation to mental health amid the current pandemic. Prolonged screen time or persistently long leisure screen time before and during the pandemic were also associated with a higher risk of psychological problems, including depressive symptoms, anxiety, and stress during the COVID-19 pandemic. In contrast, the association between change in physical activity and psychological problems did not reach statistical significance after adjusting for potential confounder. Furthermore, we found significant independent and joint associations of physical activity and leisure screen time during the pandemic with psychological problems even after adjustment for each other.

### Comparison with other studies

During the COVID-19 pandemic, lifestyle behaviors including physical activity and screen time changed significantly over a short period. There is a paucity of longitudinal research to capture this dramatic changes of lifestyle behavior and examine their association with psychological problems in children and adolescents. However, our results support previous findings from pre-pandemic cohort studies focusing on the impact of long-term change. A recent cohort study measured lifestyle behaviors from 12 to 16 years as well as depressive symptoms at 18 years, finding that persistently long screen time was associated with depressive symptoms, whereas persistently high moderate-to-vigorous-physical activity was associated with fewer depressive symptoms in adolescents [29]. The increasing use of social media and TV was also associated with depressive symptoms over four years in adolescents in another large pre-pandemic cohort study [30]. Similarly, in the present study, children and adolescents with persistently long and prolonged screen time had 2.13 and 1.48 times higher odds of having anxiety symptoms, as well as 1.70 and 1.36 times higher odds of having depressive symptoms. In addition, our study extends previous findings by revealing the significance of dramatic changes in lifestyle behaviors within a short period, which may have greater impacts on children and adolescents’ psychological conditions than long-term changes [29]. This suggests that more attention should be paid to the psychological symptoms associated with drastic short-term changes in lifestyle behaviors, especially in the context of the current recurring pandemic. Considering that depression has a high incidence and onset during childhood and adolescence, there is a need to control children and adolescents’ screen use to reduce the mental health problems during this period and the potential health risks in adulthood.

In the present study, single and joint analyses using the data obtained in the pandemic phase indicated that both increased screen time and decreased physical activity level were associated with worsening psychological conditions, even after adjustment for each other. Compared to the active students with short screen time, those with increased screen time or decreased physical activity had the highest OR, 1·95 for depressive symptoms and 1·81 for anxiety. These results suggest that long leisure screen time and insufficient moderate-to-vigorous physical activity may independently and jointly contribute to an increased risk of psychological problems during the pandemic. Currently, no published quantitative studies have investigated the joint associations of physical activity and screen time with psychological conditions during the COVID-19 pandemic. The present results are in line with previous cross-sectional studies examining the combined effect of physical activity and screen time on psychosocial difficulties or mental health in adolescents when no pandemic occurred [31–33]. Our findings highlight the importance of managing the students’ activity levels and leisure screen time to maintain a healthy mental state during the COVID-19 pandemic. Moreover, mental health intervention in children and adolescents should promote physical activity and reduce screen time at the same time to achieve optimal health benefits. Our findings supplement this part of the evidence gap and provide ideas for more effective interventions.

### Significance and implications

The present study revealed the great impact of dramatic changes in physical activity and screen time in children and adolescents over a short period, which may induce higher risks of psychological problems than long-term changes. Adequate attention should be given to these dramatic lifestyle changes because their negative influence on psychological conditions may not be mitigated spontaneously even after the COVID-19 pandemic, especially for children and adolescents with high incidence and onset of psychological problems. Today, the ongoing pandemic further increases the risk of mental health problems in children and adolescents. Thus, it is urgent to focus on physical activity and screen time simultaneously considering the independent and joint effects of these two lifestyle behaviors to resolve the major public health problem.

Because of the barrier children and adolescents face toward healthier lifestyle behaviors in the restriction of the pandemic (i.e., decreased outdoor activities), there is a need for joint family-school intervention to promote physical activity and reduce screen time in children and adolescents. On the one hand, co-activity at home with parents is one of the most effective strategies for increasing children and adolescents’ physical activity levels during lockdown [34]. On the other hand, school-level strategies, such as at-home physical education classes and scheduled breaks during online courses, are necessary [35].

### Strengths and limitations

Strengths of this study include its prospective observation under a unique circumstance during the COVID-19 pandemic, in which mandatory home quarantine resulted in a significant decrease in physical activity and an increase in screen time. This study provides evidence from a natural experiment on the effects of dramatic changes of lifestyle behaviors on mental health among children and adolescents. The large population-based sample, investigation of multiple mental disorders, and consideration of multiple potential covariates are also strengths of the study. Another strength is that baseline mental health conditions were adjusted when examining the effects of changes in lifestyle behaviors on mental health, which extended previous studies that did not consider baseline mental health conditions [29, 36, 37].

The study also has several limitations. Although a large sample of children and adolescents was recruited, the possibility of non-participation bias cannot be eliminated. In addition, since the surveys were conducted just before and during the early phase of the COVID-19 pandemic, the long-term effects of physical activity and screen time on children’s and adolescents’ mental disorder remains unclear. Furthermore, although we used validated questionnaire to measure physical activity, this questionnaire was not designed to assess light-intensity activities. Another limitation is the measurement of physical activity. Future studies may apply quantitative measurements of physical activities, such as accelerometers.

On the other hand, cross-sectional design cannot establish causality. Therefore, decreased physical activity or prolonged screen time may be the results of mental health illness as well. Our study did not include information on those who are infected, but it has been thought that it is unlikely to affect our results because the proportion of infection in children and adolescents is too small. Lastly, the generalizability of the present findings to students in other countries may be limited since the race, and cultural diversity is inadequate.

## Conclusions

This prospective large population-based study found that prolonged or persistently long leisure screen time before and during the COVID-19 pandemic were significantly associated with impaired mental health outcomes among children and adolescents. The effects of increased physical activity levels and persistently high physical activity levels on mental health outcomes were not statistically significant. On the other hand, we found significant independent and joint associations of leisure screen time and physical activity with psychological problems. Overall, the present data suggest that promoting physical activity and decreasing leisure screen time during the ongoing waves of COVID-19 effectively prevent or mitigate psychological problems in children and adolescents in China. Governments, schools, health professionals, and parents may adopt the current recommendations for exercise, encourage active lifestyle behaviors, and reduce screen time to ensure their children’s and adolescents’ physical and mental well-being under the impact of COVID-19.

## Supplementary Information

**Additional file 1: eTable 1.** Characteristics of 2423 students according to physical activity or screen time level at the second survey. **eFigure 1.** Flow chart. **eFigure 2.** Change of physical activity and leisure screen time before and during the COVID-19 pandemic among children and adolescents.

## Data Availability

The dataset supporting the conclusions of this article can be made available from the corresponding author upon reasonable request.

## Abbreviations

COVID-19: Coronavirus disease 2019
GPAQ: Global Physical Activity Questionnaire
DASS21: Depressive Anxiety Stress Scales
CDI-S: Depressive Symptoms Inventory-Short Form
SASC: Social Anxiety Scale for Children
ORs: Odds ratios
CIs: Confidence intervals
