# Supplementary material for: Association of Changes of lifestyle behaviors before and during the COVID-19 pandemic with mental health: a longitudinal study in children and adolescents
Source: Int J Behav Nutr Phys Act. 2022 Jul 26;19:92. doi: 10.1186/s12966-022-01327-8 (PMC9321278; doi:10.1186/s12966-022-01327-8)

**eTable 1. Characteristics of 2423 students according to physical activity or screen time level at the second survey**

|  | **Overall** | **Physical activity** | | **Leisure screen time** | |
| --- | --- | --- | --- | --- | --- |
|  |  | Active^a^ | Inactive | Short^b^ | Long |
| Students (*n*) | 2423 | 1740 | 683 | 1675 | 748 |
| Gender |  |  |  |  |  |
| Girls | 48.8 | 49.3 | 47.4 | 48.5 | 49.5 |
| Boys | 51.2 | 50.7 | 52.6 | 51.5 | 50.5 |
| Grade |  |  |  |  |  |
| 1-3 | 23.6 | 24.1 | 22.3 | 27.4 | 15.1 |
| 4-6 | 35.4 | 35.6 | 34.9 | 36.1 | 33.8 |
| 7-9 | 41.0 | 40.2 | 42.8 | 36.4 | 51.1 |
| Educational attainment |  |  |  |  |  |
| Father |  |  |  |  |  |
| Middle school or below | 7.4 | 6.9 | 8.7 | 6.7 | 8.8 |
| High school | 82.8 | 82.3 | 83.9 | 82.3 | 84.0 |
| University/College | 7.4 | 8.4 | 5.0 | 8.1 | 5.9 |
| Master or higher | 2.4 | 2.4 | 2.3 | 2.9 | 1.3 |
| Mother |  |  |  |  |  |
| Middle school or below | 9.8 | 9.2 | 11.3 | 9.0 | 11.7 |
| High school | 84.3 | 83.8 | 85.6 | 84.6 | 83.7 |
| University/College | 4.2 | 5.3 | 1.5 | 4.5 | 3.7 |
| Master or higher | 1.7 | 1.7 | 1.5 | 1.9 | 1.0 |
| Family income (CNY) |  |  |  |  |  |
| <100,000 | 12.0 | 10.6 | 15.4 | 11.0 | 14.2 |
| 100,000 to 200,000 | 29.4 | 28.5 | 31.7 | 28.7 | 30.9 |
| >200,000 to 400,000 | 33.3 | 34.5 | 30.1 | 34.3 | 30.9 |
| >400,000 | 16.3 | 17.1 | 14.5 | 17.1 | 14.6 |
| No answer | 9.0 | 9.3 | 8.3 | 8.8 | 9.4 |
| Psychological problems |  |  |  |  |  |
| Depression | 18.0 | 15.5 | 24.3 | 16.1 | 22.3 |
| Anxiety | 23.0 | 20.5 | 29.6 | 20.0 | 29.8 |
| Stress | 14.0 | 12.4 | 18.2 | 11.6 | 19.4 |

Data are shown as % unless otherwise specified.

Children with missing or invalid data of grade were excluded in model 1 (n=1). Children with missing or invalid data of parental education or income were further

excluded in models 2 and 3 (n=121)

^a^ Inactive was defined as no moderate- to vigorous-intensity physical activity (0 min per week), whereas active was defined as some moderate- to vigorous-intensity physical activity (>0 min per week).

^b^ Short time was defined as ≤2 hours per day whereas long time was defined as >2 hours per day.

**eFigure 1. Flow chart**


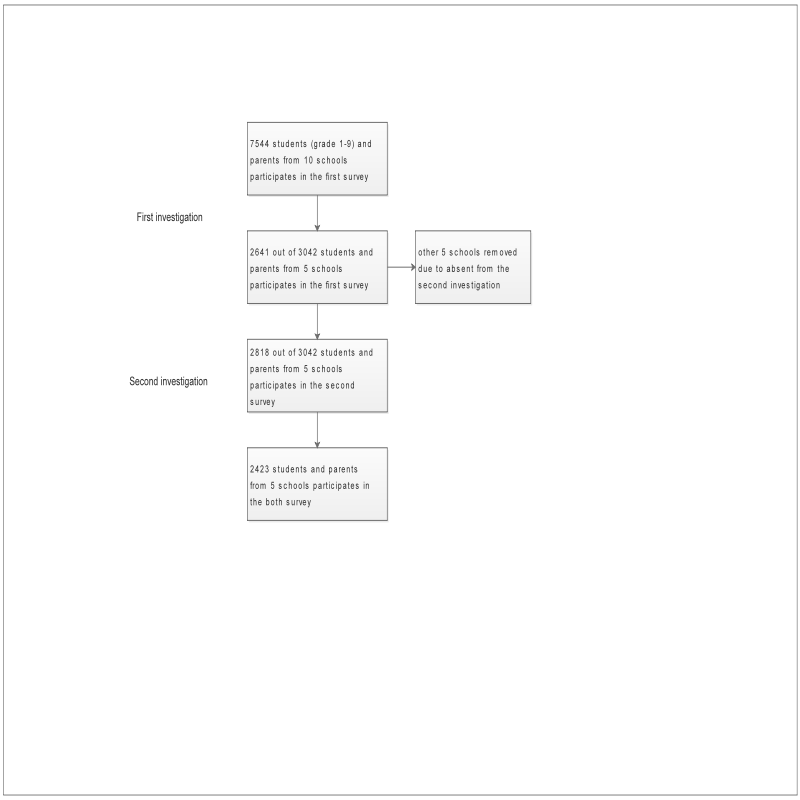


**eFigure 2. Change of physical activity and leisure screen time before and during the COVID-19 pandemic among children and adolescents**


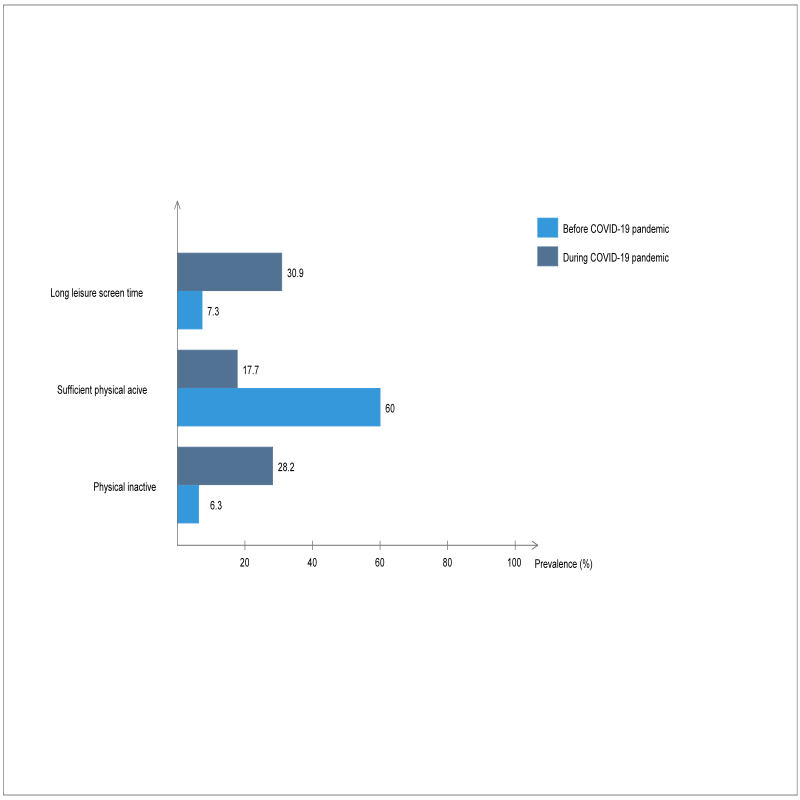

Supplement: Supplementary file 1 — Additional file 1: eTable 1. Characteristics of 2423 students according to physical activity or screen time level at the second survey. eFigure 1. Flow chart. eFigure 2. Change of physical activity and leisure screen time before and during the COVID-19 pandemic among children and adolescents. [file 12966_2022_1327_MOESM1_ESM.docx]
